# Associations of Systemic Immune-Inflammation Index and Hematological Markers with Symptom Burden and Radiological Stage in Sarcoidosis

**DOI:** 10.3390/diagnostics16132082

**Published:** 2026-07-02

**Authors:** Ezgi Erdem Türe, Berna Akıncı Özyürek, Fulsen Bozkuş, Nilgün Yilmaz Demirci, Celal Satıcı, Ayshan Mammadova, Gözde Kalbaran Kismet, Zeynep Erayman Ozen, Onur Yazıcı, Şule Taş Gülen, Burcu Akkök, Ayşegül Erinç, Nevin Fazlıoğlu, Pelin Pınar Deniz, Pınar Yıldız Gülhan, Yasemin Söyler, Aysun Şengül

**Affiliations:** 1 Department of Pulmonology, Etlik City Hospital, 06170 Ankara, Turkey; 2 Department of Pulmonology, Atatürk Sanatoryum Training and Research Hospital, 06290 Ankara, Turkey; 3 Department of Pulmonology, Faculty of Medicine, Kahramanmaraş Sütçü İmam University, 46000 Kahramanmaraş, Turkey; 4 Department of Pulmonology, Faculty of Medicine, Gazi University, 06560 Ankara, Turkeyaysenmammadova665@gmail.com (A.M.); 5 Department of Pulmonology, Yedikule Chest Diseases and Thoracic Surgery Training and Research Hospital, 34020 Istanbul, Turkey; celalsatici@yahoo.com (C.S.);; 6 Clinic of Pulmonary Medicine, Sultan 2. Abdulhamid Han Training and Research Hospital, 34668 Istanbul, Turkey; gozde468@gmail.com; 7 Department of Pulmonology, Faculty of Medicine, Aydın Adnan Menderes University, 09010 Aydın, Turkey; 8 Department of Pulmonology, Faculty of Medicine, Namık Kemal University, 59030 Tekirdağ, Turkey; 9 Department of Pulmonology, Faculty of Medicine, Çukurova University, 01790 Adana, Turkey; 10 Department of Pulmonology, Faculty of Medicine, Düzce University, 81620 Düzce, Turkey; 11 Department of Pulmonology, Faculty of Medicine, Sakarya University, 54290 Sakarya, Turkey

**Keywords:** sarcoidosis, systemic immune-inflammation index, neutrophil-to-lymphocyte ratio, platelet-to-lymphocyte ratio, red cell distribution width, mean platelet volume

## Abstract

**Background/Objectives:** Sarcoidosis is a multisystem inflammatory disease characterized by heterogeneous clinical manifestations and variable disease severity. Hematological inflammatory markers, including the neutrophil-to-lymphocyte ratio (NLR), platelet-to-lymphocyte ratio (PLR), mean platelet volume (MPV), red cell distribution width (RDW), and systemic immune-inflammation index (SII), have recently attracted attention as accessible indicators of systemic inflammation in sarcoidosis. This study aimed to evaluate radiological stage, symptom burden, pulmonary function parameters, and hematological markers in patients with sarcoidosis. **Methods:** This retrospective multicenter cohort study included histopathologically confirmed sarcoidosis patients from 10 centers. Demographic characteristics, clinical manifestations, pulmonary function test parameters, serum angiotensin-converting enzyme (ACE) levels, and hematological inflammatory markers (MPV, RDW, NLR, PLR, SII) were evaluated. Patients were categorized according to radiological stage (stage 0–I vs. stage II–IV), symptomatic status, and symptom burden (<3 vs. ≥3 symptoms). **Results:** Among 458 patients included in the study, stage I (47.8%) and stage II (46.9%) disease were the predominant radiological presentations. Patients with stage II–IV disease were older and demonstrated significantly lower forced expiratory volume in one second (FEV1), forced vital capacity (FVC), FEV1/FVC ratio, and diffusing capacity for carbon monoxide (DLCO) values together with higher serum ACE levels compared with stage 0–I disease. Dyspnea, weight loss, and extrapulmonary involvement were more frequent in stage 2–4 disease. No significant associations were observed between radiological stage and NLR, PLR, or SII values. MPV and RDW values differed significantly between symptomatic and asymptomatic patients. Patients with ≥3 symptoms demonstrated significantly lower pulmonary function parameters together with statistically significant higher NLR, PLR, and SII values. In multivariate analyses, only the SII demonstrated an independent association with a high symptom burden. **Conclusions:** Serum ACE levels were associated with advanced radiological stage, whereas hemogram-derived inflammatory indices, particularly the NLR, PLR, and SII, were associated with symptom burden rather than radiological stage in sarcoidosis and may reflect symptomatic inflammatory activity.

## 1. Introduction

Sarcoidosis is an inflammatory disease of unknown etiology characterized by the formation of non-caseating granulomas in affected tissues [1]. It is a multisystem disorder that can involve nearly any organ; however, the lungs and intrathoracic lymph nodes are most frequently affected, with pulmonary involvement observed in more than 90% of patients. Radiologically, bilateral hilar lymphadenopathy is the most characteristic finding [2]. Disease staging is primarily based on chest radiographic findings [3], with stages I and II being the most frequently observed stages [4,5].

Sarcoidosis demonstrates highly heterogeneous clinical manifestations depending on the extent and pattern of organ involvement. Patients commonly present with respiratory complaints such as cough and dyspnea, alongside extrapulmonary manifestations including lupus pernio and ocular involvement. Fatigue is another prevalent and often underrecognized symptom that may significantly impair quality of life independent of specific organ involvement [1].

Serum angiotensin-converting enzyme (ACE) has long been investigated as a biomarker in patients with sarcoidosis for many years [6]. In recent years, hematological inflammatory markers derived from peripheral blood parameters have increasingly attracted attention as accessible indicators of systemic inflammation. These include the red cell distribution width (RDW), mean platelet volume (MPV), neutrophil-to-lymphocyte ratio (NLR), platelet-to-lymphocyte ratio (PLR), and the systemic immune-inflammation index (SII). Previous studies have evaluated these markers in relation to healthy controls, disease activity, symptomatic disease, disease progression, and treatment response in sarcoidosis [7,8,9,10]. However, the relationship between peripheral blood parameters and sarcoidosis has not yet been clearly established. Therefore, the present study aimed to evaluate radiological stage, symptom burden, pulmonary function parameters, and inflammatory markers in patients with sarcoidosis.

## 2. Material and Methods

For this study, ethical approval was obtained from the local ethics committee (approval number: 2026/06, date: 26 March 2026). The study was conducted in accordance with the principles of the Declaration of Helsinki.

This study was designed as a retrospective multicenter cohort study with the participation of investigators from the Turkish Respiratory Society Early Career Researchers Group (GEAK). Data were collected using a standardized case report form across all participating centers. The study included patients from 10 centers, with center contributions ranging from 15 to 83 patients. All participants were recruited from centers across Turkey and were Turkish citizens. However, information regarding ethnic or racial background was not systematically collected and therefore could not be analyzed. Patients evaluated between May 2012 and May 2022 were screened through hospital information management systems at the participating centers.

Sarcoidosis diagnosis was established based on compatible clinical and radiological findings together with histopathological confirmation and exclusion of alternative causes of granulomatous inflammation. Histopathological confirmation was defined as the presence of non-caseating epithelioid cell granulomas obtained by tissue sampling. No patients were excluded or included on the basis of a revised histopathological diagnosis. Only patients fulfilling all diagnostic criteria for sarcoidosis following comprehensive clinical, radiological, and histopathological evaluation were included in the study. Patients lacking histopathological confirmation or not meeting the diagnostic criteria for sarcoidosis were excluded.

Chest radiographs and, when available, chest computed tomography (CT) images were retrospectively reviewed by the investigators. Radiological staging was assigned according to the Scadding classification system, based primarily on chest radiographic findings, while available CT images were used to support radiological interpretation and stage determination. According to this classification, stage I was defined as bilateral hilar lymphadenopathy, stage II as bilateral hilar lymphadenopathy with pulmonary infiltrates, stage III as pulmonary infiltrates without hilar lymphadenopathy, and stage IV as pulmonary fibrosis [3]. Asymptomatic pulmonary sarcoidosis was defined as the incidental detection of typical bilateral hilar lymphadenopathy with or without parenchymal involvement on chest radiography or computed tomography performed for routine evaluation or unrelated reasons.

Demographic characteristics (age and sex), clinical features, radiological findings, complete blood count parameters, serum ACE levels, pulmonary function test parameters, and diffusing capacity for carbon monoxide (DLCO) values obtained at the time of sarcoidosis diagnosis were recorded. To ensure consistency across participating centers, data were collected using a standardized case report form that included the systematic assessment of eight predefined symptoms (fever, arthralgia/arthritis, dyspnea, cough, fatigue, weight loss, night sweats, and erythema nodosum). To minimize treatment-related effects on inflammatory and laboratory parameters, untreated patients were included, and baseline blood parameters obtained during clinically stable periods without active infection were analyzed. The presence and type of extrapulmonary organ involvement were also documented.

NLR was calculated as neutrophil count divided by lymphocyte count. PLR was calculated as platelet count divided by lymphocyte count. SII was calculated using the following formula: platelet count × neutrophil count/lymphocyte count.

### Statistical Analysis

Descriptive variables were expressed as numbers and percentages for categorical variables and as mean ± standard deviation or median (25th–75th percentile) for continuous variables, as appropriate. Comparisons between categorical variables were performed using the chi-square test. Because the majority of patients were classified as stage I or II and the numbers of patients in stages 0, III, and IV were limited, patients were additionally grouped as stage 0–I and stage II–IV to improve statistical power and facilitate meaningful comparisons. For symptom burden analysis, patients were categorized into two groups according to the number of symptoms present at diagnosis: those with fewer than three symptoms and those with three or more symptoms. This categorization was chosen to distinguish patients with lower and higher symptom burden while maintaining adequate sample sizes and statistical power for group comparisons.

The normality of continuous variables was assessed using the Shapiro–Wilk test. Comparisons between groups were performed using the independent samples *t*-test for normally distributed variables and the Mann–Whitney U test for non-normally distributed variables. To further evaluate factors associated with high symptom burden, univariate and multivariable logistic regression analyses were performed using the presence of three or more symptoms at diagnosis as the dependent variable. Odds ratios (ORs) and 95% confidence intervals (CIs) were calculated. Because the inflammatory indices NLR, PLR, and SII are biologically related and may exhibit collinearity, separate multivariable logistic regression models were constructed for each index. All multivariable models were adjusted for age, sex, radiological stage, and DLCO. All statistical analyses were performed using the IBM SPSS Statistics for Windows, Version 26.0 (IBM Corp., Armonk, NY, USA). A *p*-value of <0.05 was considered statistically significant.

## 3. Results

A total of 458 patients with sarcoidosis were included, 313 (68.3%) of whom were female. The mean age of the cohort was 47.76 ± 13.4 years. The most common radiological stages were stage I (47.8%) and stage II (46.9%) disease. Seventy-five patients were asymptomatic at diagnosis. The most common presenting symptoms were cough (54.5%), dyspnea (38.6%), and fatigue (36%). Overall, 40.2% of patients had more than two symptoms, while 25.3% had three symptoms at presentation. Extrapulmonary involvement was observed in 125 patients (27.3%), with skin (11.4%) and ocular involvement (9.6%) representing the most frequent extrapulmonary manifestations (Table 1).

**Table 1:** Clinical and radiological characteristics of patients with sarcoidosis.

Variable	Overall (*n* = 458)	Variable	Overall (*n* = 458)
**Age, years (mean ± SD)**	47.76 ± 13.4	**Number of symptoms, *****n*** **(%)**	
**Female sex, *****n*** **(%)**	313 (68.3)	0 symptoms (asymptomatic)	75 (16.4)
**Radiological stage, *****n*** **(%)**		1 symptom	36 (7.9)
Stage 0	10 (2.2)	2 symptoms	184 (40.2)
Stage I	219 (47.8)	3 symptoms	116 (25.3)
Stage II	215 (46.9)	4 symptoms	33 (7.2)
Stage III	11 (2.4)	5 symptoms	10 (2.2)
Stage IV	3 (0.7)	6 symptoms	4 (0.9)
**Symptoms, *****n*** **(%)**		**Extrapulmonary involvement, *****n*** **(%)**	125 (27.3)
Fever	33 (7.3)	Ocular involvement	44 (9.6)
Arthralgia/arthritis	85 (18.8)	Cutaneous involvement	52 (11.4)
Dyspnea	175 (38.6)	Peripheral Lymph node involvement	2 (0.6)
Cough	247 (54.5)	Cardiac involvement	1 (0.2)
Fatigue	136 (36.0)	Hepatic involvement	22 (4.8)
Weight loss	25 (5.5)		
Night sweats	25 (5.5)		
Erythema nodosum	36 (7.9)		

Mean pulmonary function test values and mean neutrophil count, lymphocyte count, platelet count, MPV, RDW, serum ACE level, as well as NLR, PLR, and SII values are presented in Table 2.

When patients were grouped according to radiological stage, patients with stage II–IV disease (49.17 ± 14.43 years) were significantly older than those with stage 0–I disease (46.36 ± 12.19 years) (*p* = 0.025). Dyspnea and weight loss were significantly more frequent in patients with stage II–IV disease (*p* = 0.003, *p* = 0.026, respectively). The frequency of most symptoms was comparable between patients with stage 0–I and stage II–IV disease, whereas extrapulmonary involvement was significantly more common in the stage II–IV group (*p* = 0.001).

Pulmonary function parameters were significantly associated with radiological stage. Patients with stage II–IV disease had significantly lower forced expiratory volume in one second (FEV1) (*p* = 0.027), FEV1% (*p* < 0.001), forced vital capacity (FVC) (*p* = 0.024), FVC% (*p* = 0.001), FEV1/FVC ratio (*p* = 0.039), and DLCO % values (*p* < 0.001) compared with patients with stage 0–I disease.

Serum ACE levels were also significantly higher in advanced-stage disease (*p* = 0.001). However, no statistically significant differences were observed between radiological stages regarding the neutrophil count, lymphocyte count, platelet count, NLR, PLR, or SII (Table 3). Detailed symptom distribution, extrapulmonary involvement, pulmonary function test results, and laboratory parameters according to radiological stage are shown in Supplementary Table S1.

Comparison of symptomatic and asymptomatic patients demonstrated no significant differences regarding age, sex distribution, radiological stage groups, or overall extrapulmonary involvement. However, ocular involvement was significantly more frequent in symptomatic patients than in asymptomatic patients (*p* = 0.026). In addition, MPV and RDW values differed significantly between symptomatic and asymptomatic patients (*p* < 0.001 and *p* = 0.001, respectively).

When stratified according to symptom burden, patients reporting three or more symptoms were significantly older than those with fewer than three symptoms (*p* = 0.016). Higher symptom burden was also associated with a greater frequency of advanced radiological disease (stage II–IV), ocular involvement, and cutaneous involvement (*p* = 0.005, *p* = 0.035, and *p* = 0.011, respectively).

Pulmonary function analysis revealed significantly lower FEV1% (*p* = 0.039), FEV1/FVC ratio (*p* = 0.045), and DLCO% (*p* = 0.041) in patients with ≥3 symptoms compared with those with fewer symptoms. Among laboratory findings, platelet count was significantly higher in patients with ≥3 symptoms than in those with fewer than three symptoms (*p* = 0.023). Inflammatory indices including the NLR, PLR, and SII were also significant differences in patients with greater symptom burden (*p* = 0.020, *p* = 0.049, and *p* = 0.002, respectively). In contrast, MPV, RDW, and serum ACE levels did not significantly differ according to symptom burden (Table 4).

In univariate analyses, older age (OR 1.018, 95% CI 1.003–1.033, *p* = 0.017), advanced radiological stage (stage II–IV vs. stage 0–I; OR 1.745, 95% CI 1.185–2.571, *p* = 0.005), and lower DLCO values (OR 0.988, 95% CI 0.977–1.000, *p* = 0.043) were significantly associated with high symptom burden.

To evaluate the independent effects of inflammatory markers while avoiding multicollinearity, three separate multivariable logistic regression models were constructed including the PLR, NLR, and SII, respectively, together with age, sex, radiological stage, and DLCO. In the PLR model, none of the variables remained independently associated with high symptom burden, although the PLR demonstrated a borderline association (OR 1.002, 95% CI 1.000–1.005, *p* = 0.079). Similarly, in the NLR model, no significant independent association was observed for NLR (OR 1.066, 95% CI 0.948–1.198, *p* = 0.288) or for any of the other covariates. In the SII model, the SII demonstrated a borderline independent association with high symptom burden (OR 1.000, 95% CI 1.000–1.000, *p* = 0.046). Univariate and multivariate logistic regression analyses were presented in Table 5.

## 4. Discussion

Our study demonstrated that stage I and II disease represented the most common radiological presentations in patients with sarcoidosis. Patients with advanced radiological stage were older. Overall, cough, dyspnea, and fatigue were the most frequently reported symptoms. In addition, FEV1, FVC, FEV1/FVC, and DLCO values were significantly lower in patients with advanced-stage disease. However, no significant association was observed between radiological stage and asymptomatic status. Although extrapulmonary involvement was more common in advanced-stage patients, the overall frequency of extrapulmonary involvement did not significantly differ between symptomatic and asymptomatic patients. Nevertheless, ocular involvement was significantly more frequent among symptomatic patients. Serum ACE levels were significantly higher in advanced-stage disease, whereas MPV and RDW values differed significantly between symptomatic and asymptomatic patients. Notably, patients with three or more symptoms demonstrated worse pulmonary function parameters together with significant higher NLR, PLR, and SII levels.

Sarcoidosis most commonly presents with radiological stage I and II disease and is frequently symptomatic at diagnosis. Although a proportion of patients may remain asymptomatic, pulmonary involvement predominates, with cough and dyspnea representing the most common presenting symptoms [4,11]. In addition to respiratory manifestations, extrapulmonary involvement constitutes an important component of the disease spectrum. Constitutional symptoms including fever and arthralgia, granulomatous cutaneous lesions, ocular involvement, and fatigue are commonly observed in patients with sarcoidosis [12]. Fatigue, in particular, is a prominent and often underrecognized manifestation that may significantly impair quality of life and has been reported in up to 90% of patients in some series [13,14]. The skin, eyes, liver, and peripheral lymph nodes are among the most frequently involved extrapulmonary organs [11,15,16,17].

Previous studies have suggested that extrapulmonary manifestations, particularly constitutional symptoms, fatigue, cutaneous lesions, and ocular involvement, are more frequently observed in symptomatic patients [18]. However, the occurrence of extrapulmonary involvement in asymptomatic patients further highlights the systemic nature of sarcoidosis. Moreover, Judson et al. demonstrated that patients with stage I sarcoidosis were significantly more likely to be asymptomatic than those with more advanced radiological stages [18]. Consistent with the literature, stage I and II disease represented the predominant radiological presentations in our cohort. The relatively low frequency of stage III and IV disease may partly be explained by the inclusion of only histopathologically confirmed cases. Patients with stage I–II disease are more likely to undergo diagnostic procedures such as EBUS-TBNA because of prominent mediastinal lymphadenopathy, resulting in a higher likelihood of histopathological confirmation. Consequently, the study population may have been enriched with stage I–II patients, introducing a degree of selection bias and potentially limiting the generalizability of the findings to the broader sarcoidosis population. Similarly, cough, dyspnea, and fatigue were the most common clinical manifestations in our cohort, whereas cutaneous and ocular involvement represented the most frequent extrapulmonary manifestations. Although dyspnea, weight loss, and extrapulmonary involvement were more common in patients with advanced-stage disease, no significant association was observed between radiological stage and symptomatic status. Furthermore, despite the higher frequency of extrapulmonary involvement in advanced-stage disease, its overall prevalence did not differ significantly between symptomatic and asymptomatic patients. In contrast, ocular involvement was significantly more common among symptomatic patients, suggesting that certain organ involvements may contribute more substantially to clinical symptom burden than radiological extent alone.

Patients with three or more symptoms were more likely to have advanced radiological stage, ocular involvement, and cutaneous involvement. However, the relatively weak relationship between radiological stage and symptomatic status suggests that the clinical burden in sarcoidosis cannot be explained solely by the extent of pulmonary involvement. As a systemic granulomatous disease, the clinical expression of sarcoidosis is likely influenced by the overall inflammatory burden, the pattern of organ involvement, and individual host responses. Therefore, assessment of symptom burden and extrapulmonary manifestations may provide clinically relevant information beyond radiological staging alone.

Previous studies have demonstrated a close relationship between radiological stage and pulmonary function impairment in sarcoidosis, with pulmonary dysfunction becoming more prominent as disease stage advances [4]. The most common pulmonary function abnormality is a restrictive ventilatory pattern characterized by reductions in total lung capacity (TLC), vital capacity (VC), FVC and DLCO. While approximately 20% of patients with stage I disease exhibit abnormal pulmonary function tests, this rate increases to nearly 40–70% in patients with stage II–IV disease, with the most severe impairment generally observed in stage IV disease [17]. Previous studies have reported more frequent pulmonary function abnormalities among symptomatic patients; however, findings regarding the relationship between symptom status and pulmonary function parameters remain inconsistent [12,18]. Consistent with the literature, our study demonstrated significantly lower FEV1, FVC, FEV1/FVC, and DLCO values in patients with stage II–IV disease compared with stage 0–I disease. The observation that patients with a higher symptom burden also exhibited poorer pulmonary function suggests that functional impairment may contribute substantially to clinical disease expression. These findings support the association between pulmonary functional impairment and both radiological disease extent and clinical symptom burden, suggesting that patients with advanced-stage disease and greater symptom burden may require closer clinical and functional follow-up.

Previous studies evaluating hematological inflammatory markers in sarcoidosis have reported inconsistent findings regarding serum ACE levels and complete blood count parameters. One study demonstrated significantly different MPV values between sarcoidosis patients and healthy controls regardless of radiological stage, whereas no significant differences were observed for RDW and serum ACE levels between patients and healthy controls. Although serum ACE levels tended to increase with advancing radiological stage, this association did not reach statistical significance [19]. In contrast, Özsu et al. reported significantly higher baseline and follow-up RDW values in patients with stage IV sarcoidosis compared with other stages, suggesting that serial RDW measurements may be useful in predicting disease progression [7].

Several studies have demonstrated associations between elevated NLR levels and active disease, advanced radiological stage, and poor prognosis in sarcoidosis [20,21]. Similarly, previous reports have shown significantly higher NLR and PLR values in sarcoidosis patients compared with healthy controls [8]. Yalnız et al. reported significantly higher PLR levels in patients with stage II–IV pulmonary involvement compared with stage I disease [9,21]. Other studies demonstrated that elevated NLR values were more frequent in advanced radiological stages and were associated with pulmonary symptoms, particularly cough and dyspnea [19]. Likewise, Korkmaz et al. reported significantly higher NLR and PLR levels in patients with stage II–III disease; however, these indices were not associated with spontaneous remission, treatment response, or prognosis [22]. Nevertheless, the available literature remains inconsistent, and not all studies have demonstrated significant associations between inflammatory indices and radiological stage [11].

SII, which integrates neutrophil, platelet, and lymphocyte counts, has recently emerged as a novel biomarker reflecting the balance between inflammatory response and immune status. In sarcoidosis, one study demonstrated significantly higher SII levels in stage II disease compared with stage I disease [23]. In contrast, another study failed to demonstrate a significant association between SII and radiological stage but reported a significant relationship between SII and erythema nodosum [24].

Consistent with previous reports, serum ACE levels were significantly higher in patients with stage II–IV disease in our study, supporting an association between ACE levels and radiological disease extent. In contrast, MPV and RDW values differed significantly between symptomatic and asymptomatic patients rather than between radiological stages. These findings suggest that while serum ACE may be more closely related to disease extent, MPV and RDW may better reflect symptomatic inflammatory burden and clinical disease expression in sarcoidosis.

In our study, no significant association was observed between radiological stage and NLR, PLR, or SII values. This finding may partly be related to the predominance of stage I and II patients within our cohort, potentially limiting the discriminatory capacity of these inflammatory indices across radiological stages. In contrast, patients with three or more symptoms demonstrated significantly higher NLR, PLR, and SII levels. These findings suggest that hemogram-derived inflammatory indices may be associated with clinical inflammatory burden and symptomatic disease; however, these relationships should be interpreted cautiously, as various factors, including concomitant infections, comorbidities, and ongoing treatments, may influence complete blood count parameters. In multivariable logistic regression analyses adjusted for age, sex, radiological stage, and DLCO, the associations observed in univariate analyses were largely attenuated, and neither the NLR nor the PLR remained independently associated with high symptom burden. Although the SII retained borderline statistical significance after adjustment, the effect size was minimal. Overall, these findings suggest that hemogram-derived inflammatory indices may reflect the overall inflammatory milieu and symptom burden in sarcoidosis, but their independent contribution to symptomatic disease appears limited after accounting for potential confounding factors.

The present study has several limitations. First, its retrospective design may have introduced selection and information bias. Because symptom assessment was based on medical record review, symptoms that were not documented or not specifically queried during clinical evaluation may have been recorded as absent, introducing the possibility of misclassification bias. Therefore, the observed relationships between symptom burden and inflammatory indices should be interpreted with caution. Second, the predominance of stage I and II disease and the limited number of patients with stage IV fibrotic sarcoidosis may have reduced the ability to detect differences in inflammatory markers across radiological stages and limited the representation of patients with advanced fibrotic disease. Third, treatment initiation, treatment response, and longitudinal follow-up data were not available. Consequently, the ability of inflammatory indices to reflect disease progression, treatment response, and long-term disease severity could not be evaluated. In addition, disease activity could not be assessed using standardized activity criteria, limiting the interpretation of the relationship between inflammatory markers and active disease status. Information regarding concomitant ACE inhibitor use was not systematically collected across participating centers. Therefore, the potential influence of ACE inhibitor therapy on serum ACE measurements could not be assessed and cannot be completely excluded when interpreting the ACE related findings. Finally, because this was a multicenter retrospective study, laboratory measurements were performed at different institutions using local analytical platforms and laboratory procedures. Detailed information regarding analytical methods, reference ranges, and preanalytical processes was not uniformly available across all centers. Therefore, inter-center variability in laboratory measurements may have influenced the observed values of ACE and hemogram derived inflammatory indices and should be considered when interpreting the results.

## 5. Conclusions

In conclusion, serum ACE levels were significantly higher in advanced-stage sarcoidosis, supporting its association with disease burden. Although the RDW, MPV, NLR, PLR, and SII were not associated with radiological stage, these inflammatory indices were significantly higher in patients with greater symptom burdens, suggesting that they may better reflect symptomatic inflammatory activity rather than radiological extent alone. Our findings support the potential utility of easily accessible hematological inflammatory markers as adjunctive indicators of symptomatic disease in sarcoidosis.

## Figures and Tables

**Table 2:** Baseline pulmonary function and hematological inflammatory parameters in patients with sarcoidosis.

**Pulmonary Function Tests** **Mean ± SD**	
FEV1 (L)	2.63 ± 0.91
FEV1 (%)	89.33 ± 17.81
FVC (L)	3.23 ± 1.09
FVC (%)	93.38 ± 17.89
FEV1/FVC (%)	83.75 ± 11.64
DLCO (%)	82.85 ± 20.43
**Laboratory Findings** **Median (25th–75th percentile)**	
Neutrophil count (10^3^/mm^3^)	4.21 (3.45–5.55)
Lymphocyte count (10^3^/mm^3^)	1.60 (1.21–2.05)
Platelet count (10^3^/mm^3^)	280 (236–328)
MPV (f/L)	8.9 (8.0–9.8)
RDW (%)	14.0 (13.1–16.9)
Serum ACE level (U/L)	62.5 (42.0–90.0)
NLR	2.70 (2.00–3.60)
PLR	174.8 (130.9–229.8)
SII (10^3^/mm^3^)	752.6 (514.8–1074.1)

ACE, angiotensin-converting enzyme; DLCO, diffusing capacity for carbon monoxide; FEV1, forced expiratory volume in one second; FVC, forced vital capacity; MPV, mean platelet volume; NLR, neutrophil-to-lymphocyte ratio; PLR, platelet-to-lymphocyte ratio; RDW, red cell distribution width; SII, systemic immune-inflammation index; SD, standard deviation.

**Table 3:** Comparison of demographic, clinical, pulmonary function, and laboratory findings according to radiological stage groups in patients with sarcoidosis.

Variable	Stage 0–I (*n* = 229)	Stage II–IV (*n* = 229)	*p* Value
**Age, years (mean ± SD)**	46.36 ± 12.19	49.17 ± 14.43	0.025 *
**Female sex, *****n*** **(%)**	162 (70.7)	151 (65.9)	0.27
**Symptoms, *****n*** **(%)**			
Fever	17 (7.6)	16 (7.0)	0.826
Arthralgia/arthritis	49 (21.8)	36 (15.8)	0.10
Dyspnea	70 (31.1)	105 (46.1)	0.003 *
Cough	123 (54.7)	124 (54.4)	0.95
Fatigue	59 (32.6)	77 (39.1)	0.19
Weight loss	7 (3.1)	18 (7.9)	0.026 *
Night sweats	12 (5.3)	13 (5.7)	0.86
Erythema nodosum	22 (9.8)	14 (6.1)	0.15
**Extrapulmonary involvement, *****n*** **(%)**	46 (20.1)	79 (34.5)	0.001 *
Ocular involvement	23 (10.0)	21 (9.2)	0.751
Cutaneous involvement	21 (9.2)	31 (13.5)	0.141
Peripheral lymph node involvement	2 (1.2)	0	0.141
Cardiac involvement	1 (0.4)	0	0.317
Hepatic involvement	11 (4.8)	11 (4.8)	1
**Pulmonary function tests** **mean ± SD**			
FEV1 (L)	2.67 ± 0.93	2.54 ± 0.92	0.027 *
FEV1 (%)	91.55 ± 16.93	85.24 ± 19.56	<0.001 *
FVC (L)	3.33 ± 1.12	3.13 ± 1.10	0.024 *
FVC (%)	95.96 ± 17.77	90.19 ± 19.27	0.001 *
FEV1/FVC (%)	82.23 ± 10.14	81.38 ± 10.93	0.039 *
DLCO (%)	87.01 ± 17.70	78.12 ± 22.12	<0.001 *
**Laboratory findings** **Median (25th–75th percentile)**			
Neutrophil count (10^3^/mm^3^)	4.17 (3.45–5.40)	4.27 (3.45–5.60)	0.529
Lymphocyte count (10^3^/mm^3^)	1.70 (1.30–2.04)	1.54 (1.15–2.06)	0.098
Platelet count (10^3^/mm^3^)	278 (238–323)	281 (232–338)	0.711
MPV (f/L)	8.9 (8.1–9.9)	8.8 (7.8–9.6)	0.162
RDW (%)	13.9 (13.0–18.9)	14.1 (13.3–16.2)	0.714
Serum ACE level (U/L)	57.7 (40.3–78.8)	72.0 (44.0–104.5)	0.001 *
NLR	2.65 (1.95–3.38)	2.76 (2.11–3.76)	0.063
PLR	172.8 (129.8–222.6)	177.3 (131.8–235.7)	0.227
SII (10^3^/mm^3^)	748.0 (506.1–1001.0)	753.3 (519.1–1128.0)	0.281

ACE, angiotensin-converting enzyme; DLCO, diffusing capacity for carbon monoxide; FEV1, forced expiratory volume in one second; FVC, forced vital capacity; MPV, mean platelet volume; NLR, neutrophil-to-lymphocyte ratio; PLR, platelet-to-lymphocyte ratio; RDW, red cell distribution width; SII, systemic immune-inflammation index; SD, standard deviation. * Statistically significant (*p* < 0.05).

**Table 4:** Comparison of demographic, clinical, pulmonary function, and laboratory findings according to symptomatic status and symptom burden in patients with sarcoidosis.

Variable	Asymptomatic Patients (*n* = 75)	Symptomatic Patients (*n* = 383)	*p* Value	Patients with <3 Symptoms (*n* = 295)	Patients with ≥3 Symptoms (*n* = 163)	*p* Value
**Age, years (mean ± SD)**	46.20 ± 12.91	48.07 ± 13.50	0.258	46.64 ± 13.07	49.79 ± 13.82	0.016 *
**Female sex, *****n*** **(%)**	56 (74.7)	257 (67.1)	0.198	200 (67.8)	113 (69.3)	0.736
**Stage, *****n*** **(%)**						
0	3 (4.0)	7 (1.8)	0.356	7 (2.4)	3 (1.8)	0.007 *
1	39 (52.0)	180 (47)		155 (52.5)	64 (39.3)	
2	33 (44.0)	182 (47.5)		128 (43.4)	87 (53.4)	
3	0	11 (2.9)		5 (1.7)	6 (3.7)	
4	0	3 (0.8)		0 (0)	3 (1.8)	
**Stage group, *****n*** **(%)**						
Stage 0-I	42 (56.0)	187 (48.8)	0.256	162 (54.9)	67 (41.1)	0.005 *
Stage II–IV	33 (44.0)	196 (51.2)		133 (45.1)	96 (58.9)	
**Extrapulmonary involvement, *****n*** **(%)**	19 (25.3)	106 (27.7)	0.677	74 (25.1)	51 (31.3)	0.154
Ocular involvement	2 (2.7)	42 (11)	0.026 *	21 (7.1)	23 (14.1)	0.015 *
Cutaneous involvement	12 (16.0)	40 (10.4)	0.165	26 (8.8)	26 (16.0)	0.021 *
Peripheral lymph node involvement	1 (1.3)	2 (0.5)	0.630	2 (0.7)	0 (0)	0.338
Cardiac involvement	0 (0)	1 (0.3)	0.836	0 (0)	1 (0.6)	0.356
Hepatic involvement	2 (2.7)	20 (5.2)	0.344	16 (5.4)	6 (3.7)	0.404
**Pulmonary function tests** **mean ± SD**						
FEV1 (L)	2.76 ± 0.85	2.61 ± 0.92	0.448	2.67 ± 0.93	2.59 ± 0.90	0.138
FEV1 (%)	94.04 ± 12.88	88.45 ± 18.49	0.168	90.59 ± 17.25	88.01 ± 18.38	0.019 *
FVC (L)	3.21 ± 0.97	3.24 ± 1.11	0.750	3.25 ± 1.08	3.22 ± 1.11	0.150
FVC (%)	94.73 ± 12.87	93.13 ± 18.69	0.561	94.18 ± 17.35	92.54 ± 18.49	0.067
FEV1/FVC (%)	94.20 ± 12.04	81.81 ± 10.51	0.001 *	86.41 ± 12.26	80.99 ± 10.32	0.045 *
DLCO (%)	86 ± 20.79	82.64 ± 20.42	0.510	83.51 ± 20.60	82.16 ± 20.34	0.041 *
**Laboratory findings** **Median (25th–75th percentile)**						
Neutrophil count (10^3^/mm^3^)	4.17 (3.35–5.40)	4.24 (3.50–5.57)	0.527	4.17 (3.40–5.40)	4.46 (3.60–5.77)	0.103
Lymphocyte count (10^3^/mm^3^)	1.70 (1.35–2.25)	1.60 (1.20–2.00)	0.151	1.61 (1.23–2.10)	1.57 (1.20–2.04)	0.505
Platelet count (10^3^/mm3)	286 (230–327)	277 (236–329)	0.805	272 (233–318)	289 (239–343)	0.013 *
MPV (f/L)	9.5 (8.6–10.5)	8.8 (7.9–9.6)	<0.001 *	8.9 (7.9–9.8)	8.8 (8.0–9.7)	0.794
RDW (%)	15.2 (13.2–37.2)	13.9 (13.1–16.2)	0.011 *	13.8 (13.1–16.9)	14.2 (13.4–16.9)	0.222
Serum ACE level (U/L)	64.0 (48.0–92.2)	62.0 (41.0–90.0)	0.312	64.0 (45.0–89.0)	60.4 (38.8–92.9)	0.405
NLR	2.59 (1.83–3.43)	2.71 (2.05–3.63)	0.142	2.59 (1.95–3.43)	2.85 (2.24–3.75)	0.020 *
PLR	163.4 (114.7–212.2)	175.4 (131.7–230.0)	0.291	168.6 (126.3–223.8)	181.4 (139.4–233.2)	0.049 *
SII (10^3^/mm3)	733.9 (464.2–1038.3)	756.4 (518.1–1078.6)	0.213	706.3 (501.5–954.0)	856.3 (544.5–1197.3)	0.002 *

ACE, angiotensin-converting enzyme; DLCO, diffusing capacity for carbon monoxide; FEV1, forced expiratory volume in one second; FVC, forced vital capacity; MPV, mean platelet volume; NLR, neutrophil-to-lymphocyte ratio; PLR, platelet-to-lymphocyte ratio; RDW, red cell distribution width; SII, systemic immune-inflammation index; SD, standard deviation. * Statistically significant (*p* < 0.05).

**Table 5:** Univariate and multivariate logistic regression analyses.

	Univariate		Multivariate					
Variable	OR (95% CI)	*p*	PLR Model OR (95% CI)	*p*	NLR Model OR (95% CI)	*p*	SII Model OR (95% CI)	*p*
Age	1.018 (1.003–1.033)	0.017	1.012 (0.994–1.031)	0.194	1.011 (0.993–1.030)	0.226	1.004 (0.984–1.026)	0.674
Sex	0.932 (0.616–1.408)	0.736	1.037 (0.626–1.717)	0.888	0.997 (0.602–1.651)	0.990	1.154 (0.650–2.051)	0.625
Radiological stage (Stage 0–I vs. II–IV)	1.745 (1.185–2.571)	0.005	1.309 (0.839–2.042)	0.236	1.345 (0.865–2.092)	0.189	1.202 (0.724–1.997)	0.477
DLCO %	0.988 (0.977–1.000)	0.043	0.991 (0.980–1.003)	0.140	0.991 (0.979–1.002)	0.124	0.997 (0.985–1.010)	0.661
PLR	1.001 (0.999–1.003)	0.277	1.002 (1.000–1.005)	0.079	—	—	—	—
NLR	1.065 (0.967–1.172)	0.201	—	—	1.066 (0.948–1.198)	0.288	—	—
SII	1.000 (1.000–1.000)	0.856	—	—	—	—	1.000 (1.000–1.000)	0.046 *

OR, odds ratio; CI, confidence interval; DLCO, diffusing capacity for carbon monoxide; NLR, neutrophil-to-lymphocyte ratio; PLR, platelet-to-lymphocyte ratio; SII, systemic immune-inflammation index. * Statistically significant (*p* < 0.05).

## Data Availability

The data presented in this study are available on request from the corresponding author due to patient privacy and ethical restrictions.

## Supplementary Materials

The following supporting information can be downloaded at https://www.mdpi.com/article/10.3390/diagnostics16132082/s1, Table S1: Clinical and laboratory characteristics by radiological stage.

## Abbreviations

ACE	Angiotensin-Converting Enzyme
DLCO	Diffusing Capacity for Carbon Monoxide
EBUS-TBNA	Endobronchial Ultrasound-Guided Transbronchial Needle Aspiration
FEV1	Forced Expiratory Volume in One Second
FVC	Forced Vital Capacity
MPV	Mean Platelet Volume
NLR	Neutrophil-to-Lymphocyte Ratio
PLR	Platelet-to-Lymphocyte Ratio
RDW	Red Cell Distribution Width
SD	Standard Deviation
SII	Systemic Immune-Inflammation Index
TLC	Total Lung Capacity
VC	Vital Capacity
