# Supplementary material for: Associations of Systemic Immune-Inflammation Index and Hematological Markers with Symptom Burden and Radiological Stage in Sarcoidosis
Source: Diagnostics (Basel). 2026 Jul 2;16(13):2082. doi: 10.3390/diagnostics16132082 (PMC13361291; doi:10.3390/diagnostics16132082)
Supplement: Supplementary file 1 [file diagnostics-16-02082-s001.zip › diagnostics-4357341-supplementary.pdf]

Supplementary Table S1. Clinical and laboratory characteristics by radiological stage.

Variable	Stage 0 (*n* = 10)	Stage 1 (*n* = 219)	Stage 2 (*n* = 215)	Stage 3 (*n* = 11)	Stage 4 (*n* = 3)
**Age, years (mean ± SD)**	41.80 ± 10.42	46.57 ± 12.24	49.22 ± 14.39	48.18 ± 14.88	49.00 ± 21.65
**Female sex, *n* (%)**	8 (80%)	154 (70.3%)	144 (67%)	6 (54.5%)	1 (33.3%)
**Presence of Symptoms, *n* (%)**	7 (70%)	180 (82.2%)	182 (84.7%)	11 (100%)	3 (100%)
Fever	0 (0%)	17 (7.8%)	13 (6.0%)	3 (27.3%)	0 (0%)
Arthralgia/arthritis	1 (10%)	48 (21.9%)	33 (15.3%)	3 (27.3%)	0 (0%)
Dyspnea	4 (40%)	66 (30.1%)	98 (45.6%)	5 (45.5%)	3 (100%)
Cough	2 (20%)	121 (55.3%)	114 (53.0%)	7 (63.6%)	3 (100%)
Fatigue	3 (30%)	56 (25.6%)	69 (32.1%)	5 (45.5%)	3 (100%)
Weight loss	0 (0%)	7 (3.2%)	16 (7.4%)	2 (18.2)	0 (0%)
Night sweats	0 (0%)	12 (5.5%)	11 (5.1%)	2 (18.2%)	0 (0%)
Erythema nodosum	3 (30%)	19 (8.7%)	13 (6.0%)	1 (9.1%)	0 (0%)
**Extrapulmonary involvement, *n* (%)**	5 (50%)	41 (18.7%)	73 (34.0%)	5 (45.5%)	1 (33.3%)
Ocular involvement	3 (30%)	20 (9.1%)	20 (9.3%)	1 (9.1%)	0 (0%)
Cutaneous involvement	2 (20%)	19 (8.7%)	27 (12.6%)	3 (27.3%)	1 (33.3%)
Peripheral lymph node involvement	0 (0%)	2 (0.9%)	0 (0%)	0 (0%)	0 (0%)
Cardiac involvement	0 (0%)	1 (0.5%)	0 (0%)	0 (0%)	0 (0%)
Hepatic involvement	0 (0%)	11 (5.0%)	9 (4.2%)	2 (18.2%)	0 (0%)
**Pulmonary function tests mean ± SD**					
FEV1 (L)	2.8 ± 1.00	2.68 ± 0.88	2.43 ± 0.86	2.61 ± 1.06	2.77 ± 1.27
FEV1 (%)	101.57 ± 15.83	93.35 ± 17.45	86.40 ± 19.18	79.37 ± 22.89	72.00 ± 29.69
FVC (L)	3.43 ± 1.08	3.32 ± 1.06	3.03 ± 1.04	3.20 ± 1.03	3.68 ± 1.28
FVC (%)	101.75 ± 17.32	97.39 ± 17.15	91.91 ± 19.61	84.55 ± 23.39	80.50 ± 23.33
FEV1/FVC (%)	82.40 ± 3.42	82.66 ± 10.01	80.70 ± 9.90	76.89 ± 15.40	81.00 ± 19.79
DLCO (%)	79.00 ± 22.18	87.77 ± 16.92	81.15 ± 19.93	63.75 ± 24.78	53.50 ± 10.60
**Laboratory findings Median (25th–75th percentile)**					
Neutrophil count ($10^3/mm^3$)	3.17 (2.65-4.93)	4.20 (3.50-5.40)	4.24 (3.42-5.60)	4.96 (3.92-8.44)	4.27 (3.65-42.70)*
Lymphocyte count ($10^3/mm^3$)	1.97 (1.80-2.12)	1.62 (1.30-2.01)	1.52 (1.14-2.05)	1.73 (1.50-2.44)	1.83 (1.67-1.83)*
Platelet count ($10^3/mm^3$)	245 (192-314)	280 (131-515)	274 (231-337)	312 (285-400)	337 (241-337)*
MPV (f/L)	8.35 (7.82-9.70)	9.00 (8.10-9.00)	8.80 (7.80-9.67)	9.20 (8.7-9.5)	9.3 (9.1-9.3)*
RDW (%)	13.80 (12.42-15.17)	13.90 (13.00-20.10)	14.00 (13.20-16.20)	14.29 (13.50-16.00)	16.30 (13.60-16.30)*
Serum ACE level (U/L)	40.50 (23.95-58.08)	58.00 (41.00-79.00)	73.25 (44.5-103.50)	46.50 (26.75-117.50)	60.76 (43.00-60.76)*
NLR	1.57 (1.25-2.73)	2.67 (2.00-3.46)	2.76 (2.08-3.86)	2.59 (2.4-3.54)	2.33 (2.18-2.33)*
PLR	127.12 (94.47-180.59)	173.29 (131.17-225.73)	174.87 (131.10-240.74)	189.75 (175.40-198.64)	184.15 (131.69-184.15)*
SII ($10^3/mm^3$)	440.50 (285.00-602.50)	762.30 (534.00-762.30)	745.50 (499.66-1123.75)	1011.00 (740.00-1215.75)	797.00 (561.00-797.00)*

* Median (25th–50th percentile).
